# Lack of Peripherally Induced Tolerance to Established Skin Allografts in Immunologically Reconstituted *Scid* Mice

**DOI:** 10.1155/1992/36147

**Published:** 1992

**Authors:** T. V. Rajan, Leonard D. Shultz, Dale L. Greinert

**Affiliations:** 1 Department of Pathology University of Connecticut Health Center Farmington Connecticut 06030 USA; 2 Jackson Laboratory Bar Harbor Maine 04609 USA; 3 Division of Diabetes Department of Medicine University of Massachusetts Medical Center Worcester Massachusetts 01605 USA

**Keywords:** Peripheral tolerance, *scid* mice, skin allografts

## Abstract

The mechanism by which the antigen-specific immune system distinguishes between foreign antigens (toward which it mounts an immune response) and self-antigens (of which it is tolerant) is not completely understood. Studies using “superantigens” and transgenic mice have allowed investigations into some of the mechanisms of clonal deletion, anergy, and peripheral tolerance. In the present report, we have attempted to develop a new model system to investigate the possible mechanism(s) of peripheral tolerance to allografts. In this system, skin grafts from C57BL/6J (B6; *H*-2^b^ mice are grafted onto T- and B-lymphocyte-deficient C.B-17-*scid/scid* (*H*-2^d^; hereafter referred to as *scid*) mice. Because of their lack of functional lymphocytes, the *scid* mice readily accept the allogeneic skin grafts. After the allografts healed, the scid mice were reconstituted with T-cell-deficient fetal liver from coisogeneic C.B-17-∤/∤ mice or bone marrow from weanling congenitally athymic BALB/c-*nu/nu* (*H*-2^d^; hereafter referred to as *nude*) mice. Upon immunological reconstitution, the scid mice reiected the established B6 skin allografts, suggesting that an immune system developing in the presence of an intact peripheral skin allograft fails to develop tolerance to the peripheral allograft. This model system may be useful for the study of the mechanisms required for the induction of peripheral tolerance.


*Developmental Immunology*, 1992, Vol. 3, pp. 45–50
Reprints available directly from the publisher
Photocopying permitted by license only

© 1992 Harwood Academic Publishers GmbH
Printed in the United States of America

### Lack of Peripherally Induced Tolerance to Established Skin Allografts in Immunologically Reconstituted *Scid* Mice

T. V. RAJAN,*† LEONARD D. SHULTZ,‡ and DALE L. GREINER§†

†*Department of Pathology, University of Connecticut Health Center, Farmington, Connecticut 06030*
‡*Jackson Laboratory, Bar Harbor, Maine 04609*
§*Division of Diabetes, Department of Medicine, University of Massachusetts Medical Center, Worcester, Massachusetts 01605*

The mechanism by which the antigen-specific immune system distinguishes between foreign antigens (toward which it mounts an immune response) and self-antigens (of which it is tolerant) is not completely understood. Studies using "superantigens" and transgenic mice have allowed investigations into some of the mechanisms of clonal deletion, anergy, and peripheral tolerance. In the present report, we have attempted to develop a new model system to investigate the possible mechanism(s) of peripheral tolerance to allografts. In this system, skin grafts from C57BL/6J (B6; *H-2*$^b$) mice are grafted onto T- and B-lymphocyte-deficient C.B-17-*scid*/*scid* (*H-2*$^d$; hereafter referred to as *scid*) mice. Because of their lack of functional lymphocytes, the *scid* mice readily accept the allogeneic skin grafts. After the allografts healed, the *scid* mice were reconstituted with T-cell-deficient fetal liver from coisogeneic C.B-17- +/+ mice or bone marrow from weanling congenitally athymic BALB/c-*nu*/*nu* (*H-2*$^d$; hereafter referred to as *nude*) mice. Upon immunological reconstitution, the *scid* mice rejected the established B6 skin allografts, suggesting that an immune system developing in the presence of an intact peripheral skin allograft fails to develop tolerance to the peripheral allograft. This model system may be useful for the study of the mechanisms required for the induction of peripheral tolerance.

KEYWORDS: Peripheral tolerance, *scid* mice, skin allografts.

### INTRODUCTION

Over the past few years, two major technical advances have allowed the issue of self-tolerance to be examined in greater detail. First has been the observation that significant percentages of T lymphocytes are responsive to a group of "superantigens" including those encoded by the major histocompatibility complex (MHC) I-E genes, minor lymphocyte stimulatory (Mls) loci, and certain bacterial toxins (for reviews, see Webb and Sprent, 1989; Blackman et al., 1990; Kisielow et al., 1991). This has had a major impact on the understanding of tolerance to antigens expressed in the thymus by cells of the lymphoid/bone marrow lineage and suggests that there are at least two distinct mechanisms, clonal deletion and clonal anergy, by which T lymphocytes with the potential to be autoaggressive are inactivated. Second has been the development of transgenic mice useful in the study of the establishment of tolerance to immunogens that are expressed in a tissue-specific manner, particularly by solid tissues of nonlymphoid nature (MacDonald, 1989; Allison et al., 1990; Hanahan, 1990; Lo, 1990; Lo et al., 1991). Observations in transgenic mice have supported the concept that this form of antigen presentation by non-professional antigen-presenting cells (APC) results in clonal anergy and the induction of peripheral tolerance.

In the present study, we sought to develop a strategy to examine the development of tolerance to extrathymically expressed alloantigens by solid tissues, where more than one cell type might express the alloantigen. Ideally, we

*Corresponding author.

TABLE 1

Immunological Reconstitution of *scid* Mice with C.B-17-+/+ Fetal Liver[a]

| Group | Spleen | | | Thymus | | |
|---|---|---|---|---|---|---|
| | Cell number ($\times10^{-6}$) | CD3 | sIg | Cell number ($\times10^{-6}$) | CD3 | sIg |
| A (*n*=2) | 54.5 | 60.1 | 14.1 | 81.5 | 37.8 | 0.7 |
| B (*n*=5) | 37.2±8.0 | 32.9±13.0 | 21.6±13.3 | 33.2±14.3 | 28.0±10.1 | 3.5±2.8 |
| C (*n*=5) | 5.6±4.4 | 1.2±1.0 | 1.5±1.2 | <1 | N.D. | N.D. |
| D (*n*=4) | 9.1±6.1 | 28.5±8.0 | 15.6±4.9 | 5.6±7.6 | 19.7±3.9 | 3.8±0.9 |
| BALB/c (*n*=5) | 81.0±31.9 | 39.1±11.7 | 46.2±11.5 | 44.7±16.3 | 27.5±6.5 | 1.4±0.5 |

[a]A total of five *scid* mice in group A with established B6 allografts were injected i.v. with 10x10[6] C.B.-17-+/+ day-16 gestation fetal liver cells 4 weeks after skin grafting. Two were phenotyped for evaluation of immunological reconstitution at necropsy. The *scid* mice in group B were injected with C.B-17-+/+ fetal liver 2 weeks prior to B6 skin grafting. The *scid* mice in group C were grafted with B6 skin allografts with no further manipulation. The B6 skin grafted *scid* mice in group D were injected with 10x10[6] C.B-17-+/+ adult spleen cells 4 weeks after skin grafting. The BALB/c controls were 12–16 weeks of age. The relative proportion of $CD3^+$ and surface-immunoglobulin-positive ($sIg^+$) lymphocytes was determined at necropsy by flow cytometry.

wanted to establish an experimental model that would (1) permit manipulation of the immune system during its development, (2) allow induction of tolerance to various target tissues and organs, and (3) allow the utilization of the unique transgenic and mutant mice that are now available. Our basic experimental design exploits the C.B-17-*scid*/*scid* (*H-2*$^d$) mutant mouse that lacks functional T and B lymphocytes. Because such a mouse readily accepts allografts, we could transplant allogeneic tissues, allow them to heal in place, and then immunologically reconstitute the host. Our results indicate that an immune system developing in the presence of foreign skin allografts expressing both allo-class I and -class II MHC antigens is sensitized to and rejects the skin allografts. This basic model system should allow the dissection of the developing immune system and the target organs and tissues to allow determination of the parameters that may be important in the induction of peripheral tolerance.

### RESULTS

In the first series of experiments, we transplanted full-thickness skin grafts from a C57BL/6J mouse (*H-2*$^b$) on the flanks of *scid* mice. These allografts were readily accepted and allowed to heal. Four weeks after skin grafting, we injected $10\times10^6$ C.B-17-+/+ fetal liver cells into a group of mice with established B6 skin allografts (group A) and into ungrafted *scid* mice that received B6 skin allografts 2 weeks after reconstitution (group B). A group of unreconstituted *scid* mice that were grafted with B6 skin were not manipulated further (Group C) and a group of *scid* mice with established B6 skin allografts received $10\times10^6$ C.B-17-+/+ spleen cells (group D) 4 weeks after skin grafting. Skin-graft survival was followed in all four groups. As expected, mice in groups A, B, and D were immunologically reconstituted, and developed both T ($CD3^+$) and B ($sIg^+$) lymphocytes (Table 1). In contrast, unreconstituted *scid* mice with established B6 skin allografts (group C) had no detectable T or B lymphocytes.

The B6 skin grafted onto unreconstituted *scid* mice persisted until the time of necropsy (5/5) (Table 2). Skin grafts placed on group B mice (i.e., *scid* mice that had been immunologically reconstituted before allografts were placed) were

TABLE 2
Median survival Times (MST) of Allogeneic C57BL/6J (H-2$^b$) Skin Grafts on Immune Reconstituted C.B-17-*scid*/*scid* (H-2$^d$) Mice[a]

| Experiment | Treatment | MST[b] (days) |
|---|---|---|
| I | A | 44, 53, 40, 55, 45 |
| | B | <12[c] |
| | C | >70[d] |
| | D | <14[c] |
| II | A | 41, 43, 48, 43 |
| | B | <14[c] |
| | C | >70[d] |

[a]See Tables 1 and 3 for details of experimental groups I and II, respectively.
[b]The median survival time was determined by assessment of the time at which 50% of the graft was rejected.
[c]Graft rejection occurred rapidly, within 1–4 days of the first signs of rejection. All grafts in this group evidence 50% or greater rejection within this time period.
[d]Grafts remained intact throughout the observation period until necropsy.

TABLE 3
Immunological Reconstitution of *scid* Mice with BALB/c-*nu/nu* Bone Marrow.

| Group | Spleen | | | Thymus | | |
|---|---|---|---|---|---|---|
| | Cell number ($\times 10^{-6}$) | CD3 | sIg | Cell number ($\times 10^{-6}$) | CD3 | sIg |
| A[a] (*n*=4) | 80.0 | 40.1 | 51.9 | 19.0±2.7 | 78.9±5.1 | 3.3±1.6 |
| B (*n*=4) | 68.8 | 48.8 | 43.3 | 9.0 | 71.1 | <1 |
| C (*n*=5) | 5.4±3.3 | 1.2±1.8 | 1.7±1.8 | <1 | N.D. | N.D. |

[a]A total of four *scid* mice in group A with established B6 *nude* skin allografts and four *scid* mice in group B were injected i.v. with 5x10[6] BALB/c-*nu/nu* bone marrow cells; group B mice received B6 *nude* skin allografts 2 weeks later. The *scid* mice in group C received B6 *nude* skin grafts with no further manipulation. Spleens were pooled at necropsy for analysis of cytotoxic T-cell activity and phenotypic analysis. The thymuses from the four mice in group A were analyzed individually; the thymuses in group B were pooled for cell counts and phenotypic analysis. The relative proportion of each cell subset was determined by flow cytometry as in Table 1.

rejected within 12 days after engraftment (5/5). Reconstitution of B6 allograft-bearing *scid* mice with C.B-17-+/+ adult spleen cells (group D) resulted in graft rejection within 14 days (4/4). In *scid* mice with established allografts that received fetal liver (group A), grafts were rejected 47.4±6.3 days after reconstitution (5/5), suggesting a lack of tolerance induction to the peripheral allo-MHC antigens (Table 2).

Alternative explanations for the foregoing results must also be considered. The B6 skin grafts might contain passenger T lymphocytes, because they came from an immunologically normal mouse. Thus, in reconstituted *scid* mice, a mixed lymphocyte reaction might ensue within the graft, with local release of lymphokines such as interleukin-2. Essery et al. (1988) have shown this can reverse the nonresponsiveness of tolerized T lymphocytes. Also, there is a formal possibility that mature T lymphocytes with allo-specific reactivity were contained within the administered fetal liver cells. These preexisting T lymphocytes could be responsible for the skin-graft rejection, rather than T cells that developed in the *scid* thymus. To address these possibilities, we exploited available mutant mouse stocks to confirm our initial observations.

C.B-17-*scid-scid* mice were again allografted, this time using skin from B6-*nude* mice to reduce the possibility of transfer of passenger T lymphocytes. Contralateral skin grafts from BALB/c-*nude* mice were also placed on the *scid* mice. The skin grafts were accepted by the *scid* mice, and allowed to heal in place for 4 weeks. At this time, bone marrow cells from weanling BALB/c-*nude* mice were injected into B6 skin-grafted *scid* mice (group A) and into ungrafted *scid* mice (group B). Two weeks following the bone marrow injection,

TABLE 4
Anti-H-2[b] Cytotoxic T-Cell Precursor Activity in C57BL/6J (H-2[b]) *nude* Skin Grafted, Immune Reconstituted C.B-17-*scid/scid* (H-2[d]) Mice[a]

| Group | Target cell percent lysis | |
|---|---|---|
| | P815 (H-2[d]) | EL-4 (H-2[b]) |
| A | 0.3±0.5 | 20.6±1.4 |
| B | 0.9±1.2 | 28.9±1.6 |

[a]Four *scid* mice in group A with established B6 *nude* skin allografts received 5x10[6] BALB/c-*nu/nu* bone marrow cells. Four *scid* mice in group B were reconstituted with 5x10[6] BALB/c-*nu/nu* bone marrow cells and received B6 *nude* skin allografts 2 weeks later. Spleen cytotoxic T-cell precursor activity ot H-2[b] expressing EL-4 (skin-graft donor MHC haplotype) and H-2[d] expressing P815 (syngeneic MHC haplotype) chromium-51 labeled target cells were determined 2–3 weeks after final graft rejection (see Methods and Materials).

B6-*nude* and BALB/c-*nude* skin grafts were placed on contralateral flanks of each of the mice in group B. One group of skin-grafted *scid* mice was not reconstituted (group C). Skin-graft survival was followed in all three groups. At necropsy, bone marrow reconstituted *scid* mice (groups A and B) exhibited the development of both T ($CD3^+$) and B ($sIg^+$) cells, whereas unreconstituted *scid* mice (group C) had no detectable T or B lymphocytes (Table 3).

As in the first experiment, the B6-*nude* skin grafts on unreconstituted *scid* mice (group C) were still intact at necropsy (5/5); reconstituted and then grafted *scid* mice (group B) rejected the B6-*nude* skin grafts within 14 days (4/4) (Table 3). The well-healed allogeneic B6-*nude* skin grafts in group A *scid* mice were rejected 43.8+/−3.0 days after reconstitution with BALB/c-*nude* bone marrow (4/4) (Table 3). The histocompatible BALB/c-*nude* skin grafts remained intact in all mice. Furthermore, spleen cells recovered from reconstituted *scid* mice (groups A and B) demonstrated high cytotoxic T-cell precursor activity to the *H-2*$^b$ allo-MHC *in vitro* (Table 4), again suggesting a lack of tolerance to the peripherally expressed skin allograft.

### DISCUSSION

In the present study, we have demonstrated that when T cells mature in the presence of a peripheral skin allograft, tolerance does not develop. The rejection of the skin allograft does not appear to be due to the transfer of preformed alloreactive T cells, because bone marrow from athymic *nude* donors developed into lymphocytes reactive to the allo-MHC that were capable of graft rejection. However, it has been observed that *nude* mice do develop T cells as they age, although young *nude* mice are severely deficient in T cells (Holub, 1989; Vaesseen et al., 1986). Thus, although it cannot be formally excluded that transferred preformed T cells in the *nude* bone marrow inoculum contributed to the graft rejection, our use of young (4–6 weeks of age) *nude* mice as bone marrow donors reduced this possibility. In addition, the possible contribution of T lymphocytes from the skin graft itself was reduced by the use of skin from *nude* mice, and further studies in our laboratories using *scid* mice as skin-graft donors support this conclusion.

Of particular interest is the difference in our results to that observed in many of the transgenic experiments. Although many possible explanations might explain our divergent results, at least two likely possibilities readily emerge. In the transgenic studies, when an alloantigen is expressed in a strictly cell- and tissue-specific manner, the immune system of the animal is tolerant (Hanahan et al., 1989; Lo, 1990; Miller et al., 1990; Lo et al., 1991). In these mice, the transgene is present in the genome, and might be expressed at low levels in the thymus, resulting in intrathymic clonal deletion rather than induction of peripheral tolerance. However, investigators have used several sensitive assays including S1 protection and PCR to ensure that there is no detectable expression of the transgene in the thymuses of the mice. Furthermore, Lo et al. (1991) replaced the thymus and bone marrow with nontransgenic tissues, absolutely precluding endogenous thymic expression of the product of the transgene.

A second difference is that expression of the alloantigen in our model occurs in numerous cell types and in several discrete tissues, including the epidermis and dermis of the skin. It is possible that in our system, the alloreactive T cells could be given a stimulatory second signal by epidermal dendritic cells or Langerhans cells, that is, professional APCs (for review, see Steinman, 1991; also see Schuler and Steinman, 1985; Heufler et al., 1987). In contrast, in the transgenic systems of peripheral tolerance, the expression of the alloantigens is confined exclusively to that of class II-negative, nonprofessional APC. The *in vitro* tolerization model described by Schwartz et al. (1990) proposes that exposure of T cells to antigen in the absence of appropriate APCs results in tolerance rather than activation. This suggests that in transgenic mice, the antigen is presented on nonprofessional APC, resulting in the absence of an appropriate "second" signal.

Lack of professional APCs in an allograft would then be predicted to result in tolerance induction. It has been estimated that Langerhans cells turn over in the skin in about 5 weeks (Lacey and Davey, 1984). Therefore, in a preliminary experiment, we analyzed the effect of prolonged graft healing before immunological reconstitution. A C.B-17-*scid/scid* mouse with an intact C57BL/6-*scid/scid* skin graft was reconstituted 20 weeks later with BALB/c-*nu/nu* bone marrow. This time period of graft survival should have allowed Langerhans cells in the graft to turn over prior to bone marrow reconstitution. However, 8 weeks after reconstitution, the graft was rejected. This preliminary experimental result suggests that the presence (or absence) of Langerhans cells in the skin grafts may not be the sole crucial element in determining graft survival. This is consistent with the observations of Streilein et al. (1979, 1982), who used skin and cornea as grafts that differed in the presence or absence of Langerhans cells, respectively. They observed that corneal grafts were accepted by MHC class II disparate hosts, whereas identical donor-skin allografts were accepted only when the graft was pretreated to eliminate Langerhans cells. In contrast, both corneal and cutaneous allografts were rejected in MHC class I disparate combinations.

Our results are consistent with those of Besedovsky et al. (1979). They observed that *nude* mice rejected healed allografts when immunologically reconstituted with syngeneic thymic implants. More recently, Gao et al. (1991) observed a lack of tolerance induction in chimeras when newly maturing lymphocytes were exposed to MHC class II alloantigens on "nonprofessional" APCs.

Our model system, as described, now allows some of the parameters that are necessary for peripheral tolerance induction to be examined. In ongoing studies, we are investigating the induction of peripheral tolerance to MHC class I and class II deficient skin grafts and islet grafts, and we are investigating the possible role of B lymphocytes in induction of peripheral tolerance to allografts. We believe that this basic model system now can be modified to investigate and to individually dissect the effects that perturbations in lymphocyte development and repertoire generation have on peripheral tolerance induction, to determine how to induce tolerance to different organ grafts and tissues, and to investigate how alteration of the grafted tissues, either by genetic (transgenic or knockout technology) or *in vitro* pretreatment (e.g. to remove APC), effects the induction of peripheral tolerance.

### METHODS AND MATERIALS

### Mice.

C.B-17-*scid*/*scid* mice, C.B-17-+/+ mice, C57BL/6J-*nu*/*nu* mice, BALB/c-*nu*/*nu* mice, and C57BL/6J-+/+ mice were obtained from the colony maintained by LDS at the Jackson Laboratory (Bar Harbor, Maine). Because of the severe immunodeficiency of the *scid* and *nude* mice, both recipient and donor mice were maintained in microisolator cages and provided autoclaved food and water *ad libitum*. The *scid* mice were also maintained prophylactically on a combination of sulfamethoxazole and trimethoprim (Nelson et al., 1991), which prevents them from succumbing to *Pneumocystis carinii* pneumonia.

### Antibodies.

The anti-CD3 monoclonal antibody (2C11) was the kind gift of Dr. J. Bluestone (Chicago, Illinois), and was developed for immunofluorescence with mouse antihamster Ig-FITC. Surface immunoglobulin-positive (B) cells were detected with an FITC-conjugated goat antimouse IgG (heavy- and light-chain-specific). The percent positive cells were determined by flow cytometry as previously described (Nelson et al., 1991).

### Cell Suspensions.

Bone marrow cell suspensions were obtained by flushing the tibia and femur with cold medium (HEPES buffered RPMI 1640). Fetal liver was recovered from fetuses at day 17–19 of gestational development. Thymus, spleen, and fetal liver-cell suspensions were prepared by gently pressing the tissues through a 50-mesh cell sieve followed by washing in cold medium. For flow cytometry analysis, red blood cells were removed by hypotonic lysis using 0.168 molar $NH_4CL$. Cell viability was determined by exclusion of 0.1% trypan blue and was greater than 95% in all cases.

### Skin Grafting.

Skin graft donors were shaved, and approximately 2x2-cm full-thickness skin grafts were prepared and grafted onto the flanks of the *scid* mice (see text for details of specific donor and recipient groups). Skin-graft rejection was evaluated daily and the end point was defined as the day when 50% of the skin graft detached from the graft bed (Medawar, 1944; Billingham and Medawar, 1951; Klein, 1975).

### Adoptive Transfer.

Single-cell suspensions of fetal liver, bone marrow, or spleen were suspended at a concentration of $20\times10^6$/ml and 0.5 ml was injected i.v. into the tail vein of unirradiated *scid* mice.

### Cytotoxic T-Cell Precursor Assay.

This assay was performed as previously described (Woan et al., 1981). Briefly, spleen cells were recovered from immune reconstituted C.B-17-*scid*/*scid* mice. Sterile single-cell suspensions were prepared, and $3.2\times10^5$ cells were incubated with $2\times10^5$ 2000-rad irradiated C57BL/6J spleen cells in individual wells of a 96-well U-bottom microtiter plate in the presence of 6 units of exogenous IL-2 for 7 days. Following sensitization, $5\times10^4$ $^{51}$Cr-labeled EL-4 (H-$2^b$) or P815 (H-$2^d$) target cells were added to each well, and incubated for an additional 4 hours. The amount of killing, that is, the amount of released $^{51}Cr$, was determined by spinning the plates at 250 g for 5 min, harvesting the supernatant, and determining the counts in the recovered supernatant by gamma counting. The specific lysis presented is the mean±standard deviation of quadruplicate wells and was calculated as previously described (Woan et al., 1981).

### ACKNOWLEDGMENTS

We wish to acknowledge the excellent technical assistance of Ms. Pat Porte, Mrs. Anita Wayne, Ms. Kathleen Fitzgerald, and Mr. Bruce Gott. This work was supported in part by grants from the National Institutes of Health: R22-AI27773 (TVR); R01-AI30389 (LDS); R22-AI30046 (TVR, DLG and LDS), and R01-DK36024 (DLG) and by a grant from the Juvenile Diabetes Foundation, Int. (DLG).

*(Received April 2, 1992)*

*(Accepted June 2, 1992)*

### REFERENCES

Allison J., Harrison L.C., Campbell I.L., and Miller J.F. (1990). Major histocompatibility complex molecules and the beta cell: Inferences from transgenic models. Curr. Top. Microbiol. Immunol. **156:** 121–135.

Besedovsky H.O., Del R.A., and Sorkin E. (1979). Role of prethymic cells in acquisition of self-tolerance. J. Exp. Med. **150:** 1351–1358.

Billingham R.E., and Medawar P.B. (1951). The technique of free skin grafting in mammals. J. Exp. Biol. **28:** 385–402.

Blackman M.A., Kappler P., and Marrack P. (1990). The role of the T cell receptor in positive and negative selection of developing T cells. Science **248:** 1335–1341.

Essery G., Feldmann M., and Lamb J.R. (1988) Interleukin-2 can prevent and reverse antigen-induced unresponsiveness in cloned human T lymphocytes. Immunol. **64:** 413–417.

Gao E.-K., Kosada H., Surh C.D., and Sprent J. (1991). T cell contact with Ia antigens on nonhemopoietic cells *in vivo* can lead to immunity rather than tolerance. J. Exp. Med. **174:** 435–446.

Hanahan D. (1990). Transgenic mouse models of self-tolerance and autoreactivity by the immune system. Annu. Rev. Cell. Biol. **6:** 493–537.

Hanahan D., Jolicoeur C., Alpert S., and Skowronski J. (1989). Alternative self or nonself recognition of an antigen expressed in a rare cell type in transgenic mice: Implications for self-tolerance and autoimmunity. Cold Spring Harb. Symp. Quant. Biol. **2:** 821–835.

Heufler C., Koch F., and Schuler G. (1987). Granulocyte-macrophage colony-stimulating factor and interleukin-1 mediate the maturation of murine epidermal Langerhans cells into potent immunostimulatory dendritic cells. J. Exp. Med. **167:** 700–705.

Holub M. (1989). Immunology of nude mice (Boca Raton, FL: CRC Press), pp. 53–62.

Kisielow P., Swat W., Rocha B., and von Boehmer H. (1991). Induction of immunological unresponsiveness *in vivo* and *in vitro* by conventional and super-antigens in developing and mature T cells. Immunol. Rev. **122:** 69–86.

Klein J. (1975). Biology of mouse histocompatibility-2 complex (New York: Springer-Verlag), pp. 142–146.

Lacey P.E., and Davey J.M. (1984). Transplantation of pancreatic islets. Ann. Rev. Immunol. **2:** 183–198.

Lo D. (1990). Immune responses to tissue-restricted self antigens: Studies on T cell tolerance and autoimmunity to pancreatic beta cells. Curr. Top. Microbiol. Immunol. **164:** 71–94.

Lo D., Freedman J., Hesse S., Brinster R.L., and Sherman L. (1991). Peripheral tolerance in transgenic mice: Tolerance to class II MHC and non-MHC transgene antigens. Immunol. Rev. **122:** 87–102.

MacDonald H.R. (1989). Mechanisms of immunological tolerance. Science **246:** 982–984.

Medawar P.B. (1944). The behaviour and fate of skin autografts and skin homografts in rabbits. J. Anat. **78:** 176–199.

Miller J.F., Morahan G., Slattery R., and Allison J. (1990). Transgenic models of T-cell self tolerance and autoimmunity. Immunol. Rev. **118:** 21–35.

Nelson F.K., Greiner D.L., Shultz L.D., and Rajan T.V. (1991). The immunodeficient scid mouse as a model for human lymphatic filariasis. J. Exp. Med. **173:** 659–663.

Schuler G., and Steinman R.M. (1985). Murine epidermal Langerhans cells mature into potent immunostimulatory dendritic cells *in vitro*. J. Exp. Med. **161:** 526–546.

Schwartz R.H. (1990). A cell culture model for T lymphocyte clonal anergy. Science **248:** 1349–1356.

Steinman R.M. (1991). The dendritic cell system and its role in immunogenicity. Ann. Rev. Immunol. **9:** 271–296.

Streilein J.W., Lonsberry L.W., and Bergstresser P.R. (1982). Depletion of epidermal Langerhans cells and Ia immunogenicity from tape-stripped mouse skin. J. Exp. Med. **155:** 863–871.

Streilein J.W., Towes G.B., and Bergstresser P.R. (1979). Corneal allografts fail to express Ia antigens. Nature **282:** 326–327.

Vaessen L.M.B., Broekhuizen R., Rozing J., Vos J.G., and Schuurman H.-J. (1986). T-cell development during ageing in congenitally athymic (nude) rats. Scand. J. Immunol. **24:** 223–235.

Webb S.R., and Sprent J. (1989). T-cell responses and tolerance to Mls a determinants. Immunol. Rev. **107:** 141–158.

Woan M.C., McGregor D.D., and Forsum U. (1981). T cell-mediated cytotoxicity induced by Listeria monocytogenes. II. Specificity of cytolytic effector cells. J. Immunol. **127:** 2325–2330.

